# Correction to “Chemotherapy outcomes in EGFR‐TKI resistant patients with common and uncommon *EGFR* mutation: An exploratory retrospective cohort study”

**DOI:** 10.1111/1759-7714.15242

**Published:** 2024-02-20

Lin C‐Y, Hu C‐H, Hsu C‐F, Tsai J‐S, Chen C‐W, Lin C‐Y, et al. Chemotherapy outcomes in EGFR‐TKI resistant patients with common and uncommon *EGFR* mutation: An exploratory retrospective cohort study. Thorac Cancer. 2023;14(19):1857–64. https://doi.org/10.1111/1759-7714.14930

The authors have identified an error in one of the affiliations listed in the publication. The correct affiliation for Chia‐Fu Hsu should be as follows:

^2^Division of Pulmonary and Critical Care Medicine, An Nan Hospital, China Medical University, Tainan, Taiwan, ROC.

We apologize for this error.

